# Long-Term Low-Level Arsenic Exposure Is Associated with Poorer Neuropsychological Functioning: A Project FRONTIER Study

**DOI:** 10.3390/ijerph8030861

**Published:** 2011-03-15

**Authors:** Sid E. O’Bryant, Melissa Edwards, Chloe V. Menon, Gordon Gong, Robert Barber

**Affiliations:** 1 F. Marie Hall Institute for Rural and Community Health, Texas Tech University Health Sciences Center, 3601 4th St., STOP 6323, Lubbock, TX 79430, USA; E-Mail: Gordon.Gong@ttuhsc.edu; 2 Department of Neurology, Texas Tech University Health Sciences Center, 3601 4th St., STOP 8321, Lubbock, TX 79430, USA; 3 Department of Psychology, Texas Tech University, 2500 Broadway, STOP 2051, Lubbock, TX 79409, USA; E-Mails: Ml.Edwards@ttu.edu (M.E.); chloe.menon@ttu.edu (C.V.M.); 4 Department of Pharmacology and Neuroscience, Institute for Aging and Alzheimer’s Disease, University of North Texas Health Science Center, 3500 Camp Bowie Boulevard, Ft Worth, TX 76107, USA; E-Mail: Robert.Barber@unthsc.edu

**Keywords:** arsenic, chronic exposure, environmental exposure, cognition, rural health

## Abstract

Exposure to elements in groundwater (toxic or beneficial) is commonplace yet, outside of lead and mercury, little research has examined the impact of many commonly occurring environmental exposures on mental abilities during the aging process. Inorganic arsenic is a known neurotoxin that has both neurodevelopmental and neurocognitive consequences. The aim of this study was to examine the potential association between current and long-term arsenic exposure and detailed neuropsychological functioning in a sample of rural-dwelling adults and elders. Data were analyzed from 434 participants (133 men and 301 women) of Project FRONTIER, a community-based participatory research study of the epidemiology of health issues of rural-dwelling adults and elders. The results of the study showed that GIS-based groundwater arsenic exposure (current and long-term) was significantly related to poorer scores in language, visuospatial skills, and executive functioning. Additionally, long-term low-level exposure to arsenic was significantly correlated to poorer scores in global cognition, processing speed and immediate memory. The finding of a correlation between arsenic and the domains of executive functioning and memory is of critical importance as these are cognitive domains that reflect the earliest manifestations of Alzheimer’s disease. Additional work is warranted given the population health implications associated with long-term low-level arsenic exposure.

## 1. Introduction

The detrimental impact of acute high-level exposure to arsenic on health is well established; however, prior work has also documented adverse consequences of prolonged exposure to groundwater arsenic at levels below the current U.S. standard of 10 μg/L. Chronic exposure to low-levels of arsenic through drinking water is common in the U.S. where 13.6% of sampled public water-supply systems exceeded 5 μg/L and 25% exceeded 2 μg/L [1]. Such exposure has been found to increase risk for a range of diseases including hypertension, diabetes, coronary artery disease, skin melanosis, cancer, and poorer cognition [2–8]. Therefore, over 40 million Americans are at an increased risk for negative health consequences resulting from being exposed to low-levels of arsenic over the course of their lifetime [1]. In fact, it has been proposed that exposure to environmental toxins, including arsenic, has caused a “silent pandemic” in modern society that has gone undetected [9]. This circumstance has likely remained unnoticed because the neurodevelopmental and neurotoxic consequences of *in utero* exposure (along with chronic lifetime exposure) may not become evident until neuronal attrition associated with aging occurs [9]. To date, however, no prior work has been published looking at the potential impact of long-term low-level arsenic exposure on cognitive functioning among adults and elders. The purpose of this study was to take a first-step towards addressing this gap in the public health literature.

There is ample reason to hypothesize an association between chronic arsenic exposure at low levels and neuropsychological dysfunction. Inorganic arsenic at high doses is a known neurotoxin with both neurodevelopmental and neurocognitive consequences. From a neuropathological standpoint, arsenic exposure has been associated with an increase in the production of β amyloid [10], hyperphosphorylation of tau protein [11], oxidative stress [12], inflammation [13,14], endothelial cell dysfunction [15] and angiogenesis [16], all of which have been linked to cognitive dysfunction and are proposed mechanisms underlying Alzheimer’s disease [3,17–19]. In animal models, arsenic exposure has been shown to cause morphologic and neurochemical alterations in the hippocampus and other memory-related neuronal structures and expected learning and memory deficits have been noted [15,20,21]. However, the direct link between chronic low-level arsenic exposure and detailed neuropsychological status remains untested.

Despite the National Research Council’s call for epidemiological studies of the non-cancer health consequences of long-term low-level arsenic exposure [7,8], little research has been conducted to date. This is not for lack of interest; however, the methods for conducting such investigations have yet to be established. The ideal situation is one where a biomarker of chronic exposure is available, which is the case when considering lead. Bone lead level measurement via K-shell-x-ray fluorescence has been used as a biomarker of long-term exposure [22] and the correlation between such exposure and cognitive functioning has been studied [23,24]. However, a valid biomarker for long-term arsenic exposure is lacking. One method that has been utilized is to reconstruct chronic exposures according to retrospective report of the level as well as the duration of exposure. In the PHYTHONER study, Baldi and colleagues [25] have documented a detrimental impact of long-term pesticide exposures on neurocognitive functioning among French vineyard workers using this approach. In countries where high levels of arsenic exposure have been documented for decades (e.g., areas of China, Bangladesh, and Mexico), researchers have historical data regarding exposure levels that can be utilized to create models of chronic exposure. In the U.S., the state of Texas offers a very unique situation given that the Texas Water Development Board (TWDB) has been monitoring well-water levels of a large number of chemicals including arsenic, for over 15 years across the state. Additionally, we have been conducting an epidemiological study of rural health, Project FRONTIER, for several years in two West Texas counties with mean groundwater arsenic levels that are below the U.S. standard. As part of this protocol, we have collected subjects’ (1) exact residential location and (2) number of years living at that location. Therefore, combining data from the TWDB and Project FRONTIER can create estimates of current and long-term arsenic exposure. Groundwater arsenic concentration at each subject’s home can be estimated with Geographic Information System (GIS) approach (the ArcGIS program) based on the residential location’s distances to surrounding wells with known groundwater arsenic concentrations provided by TWDB. The purpose of the current study was to examine the potential association between current and long-term arsenic exposure estimated by the GIS methods and detailed neuropsychological functioning in a sample of rural-dwelling adults and elders. Based on our prior work, as well as work with children and adolescents, we hypothesized that increased low-level arsenic exposure would be significantly correlated with poorer scores in the domains of global cognition, executive functioning, memory, and language.

## 2. Experimental Section

### 2.1. Participants

**Figure 1.**
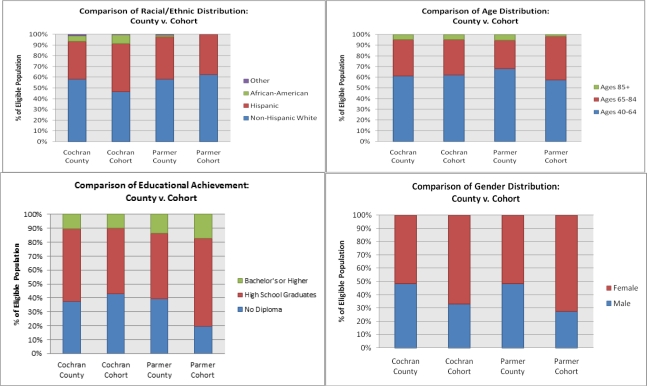
Demographic comparison of enrolled participants (Cochran Cohort & Parmer Cohort) *versus* all eligible individuals in each county (Cochran County & Parmer County) for race/ethnicity, age, education, and gender.

Data from 434 participants (133 men and 301 women) from Project FRONTIER were analyzed. Project FRONTIER is an ongoing epidemiological study of cognitive aging among rural-dwelling individuals. Project FRONTIER utilizes a community-based participatory research (CBPR) approach, which is a research methodology that involves partnering communities with scientific groups to conduct studies of human disease that is growing rapidly in terms of use and acceptance in the scientific community. CBPR is particularly useful when working with underserved communities that may not respond to classic approaches (e.g., random digit dialing, mail surveys) and is very well suited to rural health research; CBPR is supported by and recommended for rural research by the National Institute of Environmental Health Sciences [26]. In Project FRONTIER, we have spent several years establishing and maintaining our community ties through local advisory boards, presentations, hiring of local workers into the research infrastructure, and partnering with community entities for completion of parts of the research protocol (*i.e.*, blood work and medical examinations); our CBPR process for maintaining this translational research platform has been described in detail elsewhere [27]. Briefly, we partner with the local hospitals and clinics (who conduct the medical examinations and clinical labs as well as provide office space) as well as the senior citizen’s organizations. Our community recruiters, community individuals, and research staff present information about the study at community events as well as through door-to-door solicitation. The distribution of participants recruited into the study (Cochran Cohort & Parmer Cohort) as compared to all eligible individuals by county (Cochran County & Parmer County) by race/ethnicity, age, education, and gender are provided below in Figure 1. As can be seen from these figures, the demographic composition of our cohort closely resembles that of the eligible population from which they are drawn.

### 2.2. Procedures

The protocol includes a standardized medical examination, clinical labs and neuropsychological testing, as well as an interview with the participant and a brief interview with an informant. Inclusion criteria are (1) age 40 and above and (2) residing in one of the counties participating in Project FRONTIER. The two counties currently part of project FRONTIER are Cochran County and Parmer County, both located west of Lubbock, Texas, on the Texas—New Mexico border. The U.S. Census conducted in 2008 indicated that the Cochran County comprised 3,501 residents with 1,609 individuals age 40 and over (779 men and 830 women), compared to 9,639 residents for Parmer County including 3,937 individuals age 40 and over (1,906 men and 2,031 women). Additionally, twenty-two percent of Cochran County inhabitants were reported to live below the poverty level, relative to 14% reported in Parmer County. The principal economic land use in Cochran and Parmer County is large-scale irrigated farming (cotton and wheat), ranching, and oil and natural gas production. Participant recruitment is conducted by community recruiters through different means including brochures/flyers, presentations and events, as well as, in-person and/or door-to-door solicitation. All participants signed written informed consent and Project FRONTIER is conducted under an IRB approved protocol.

The neuropsychological test battery was comprised of instruments covering a range of cognitive domains. The *Mini-Mental State Examination* (MMSE) [28] is the most commonly administered psychometric screening assessment of global cognitive functioning. Since its development, there has been a wealth of literature published on the MMSE demonstrating it to be a relatively sensitive marker of dementia [29]. The *Exit Interview* (EXIT25) [30] is a well-validated global measure of executive control that covers a range of tasks including sequencing, fluency, anomalous sentence repetition, thematic perception, automatic behaviors, go-no-go and automatic behavior, among others. EXIT25 scores are significantly correlated with other validated measures of executive functioning [30]. Scores range from zero to 50 with higher scores suggestive of greater impairment; a score of 15 or greater best discriminates non-demented elderly controls from those with dementing illnesses. The *Repeatable Battery for the Assessment of Neuropsychological Status* (RBANS) [31] is a brief neuropsychological instrument that assesses multiple cognitive domains [32]. It contains 12 subtests that combine to create five indices: Attention, Language, Visuospatial/Constructional abilities, as well as Immediate and Delayed recall. The RBANS has accumulated a large amount of normative data [33] and has well established psychometric properties [34]. The *Trails Making Test* (TMTA and TMTB), is a neuropsychological instrument that measures attention, processing speed and mental flexibility, and is considered to be a sensitive marker of cognitive dysfunction and decline [35]. The Co*ntrolled Oral Word Association* Test (COWAT) [35] assesses both phonemic (FAS) and categorical (Animal Naming) verbal fluency, both of which are standard neuropsychological assessment techniques with high sensitivity to cognitive dysfunction and dementia [35].

**Figure 2.**
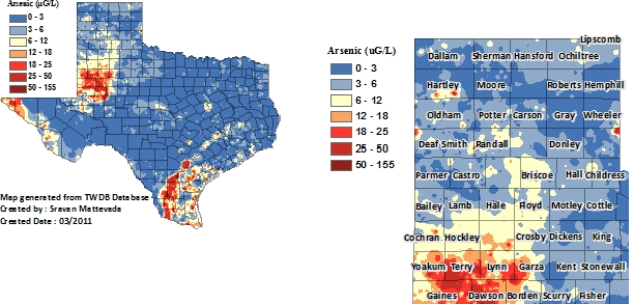
Ground water arsenic measurements form the Texas Water Development Board (TWDB).

*Determination of GIS-Arsenic*. Geographic information system (GIS) is a way of displaying and analyzing geographically referenced information. GIS-based methods are commonly used to estimate environmental exposures [3,36–39]. We used the Environmental Systems Research Institute [40] ArcGIS (release 9.2) program to plot a point for each of the 16,335 arsenic ground water measurements that are readily available from the Texas Water Development Board (TWDB) [41]. Through inverse distance weighted (IDW) interpolation, the ArcGIS software builds a three dimensional surface map from a list of points. Each point’s influence is weighted based off its distance to that section of the map, which was generated using 12 well measurements from the TWDB within the immediate geographic vicinity. Each of the study participant’s current residential address was geocoded with the ArcGIS StreetMap data. Finally, GIS-arsenic concentration was calculated by extracting the estimated arsenic value from the IDW surface at each resident’s location. The maps of arsenic concentration in Texas (left) and Texas Panhandle (right) are presented below (Figure 2). Arsenic groundwater levels are found to increase from Parmer County (15-year mean As level of 3.0 μg/L) south to Cochran County (15-year mean As value of 7.4 μg/L with a maximum of 15.6 μg/L at the “hot spot”). Additionally, the length of residential history of residents from our pilot study suggests that large portions of the community are long-term residents averaging over 30 years living in their respective communities. Combined, these data demonstrate that the two counties selected for this study provide an optimal naturalistic setting to investigate long-term low-level arsenic exposure from groundwater.

*Calculation of long-term low-level arsenic exposure.* In order to estimate long-term low-level arsenic exposure, we used readily available TWDB historical data and data from Project FRONTIER regarding current residential address as well as years living at that residence. We examined 15-years of TWDB water arsenic levels from wells within the communities of Project FRONTIER. We estimated long-term low-level exposure by multiplying current estimated arsenic levels by the number of years residing in current home. Similar methods for estimating long-term exposure have been utilized previously [42,43]. As part of a different research study, we pilot tested the comparability between observed and estimated (current) groundwater arsenic levels. We collected data from 7 rural wells and examined their actual values by an atomic fluorescence method [42,43] and compared these data with their respective GIS-estimated arsenic concentrations. GIS-estimated *versus* measured values are listed in Table 1. GIS-estimated arsenic concentrations were quite close to measured values except wells number 6 and 7, of which GIS-arsenic values are 4.6 and 4.2 μg/L higher than the measured values. However, the ranks of the two series of value are nearly identical (with the exception that rank 6 and 7 based GIS approach for well #6 and #7 are a tie by measured values).

Linear regression models were created with SPSS version 18 using raw neuropsychological test scores as outcome variables and either current or long-term arsenic exposure estimates as predictor variables. Covariates considered in the models included age, gender, education, ethnicity, language of administration, and APOE4 status (present/absent). Given that selenium is known to impact arsenic toxicity, current groundwater selenium estimates were also calculated via GIS methods and were entered into the models as a covariate. We utilized receiver operating characteristic (ROC) curves to estimate the accuracy of arsenic levels in classifying cognitive status (impairment *versus* no impairment) by reviewing the area under the ROC curve (AUC). Statistical significance was set at p < 0.05.

## 3. Results and Discussions

The mean age and education of the 434 participants was 62.12 (sd = 12.81; range = 40–96) and 10.84 (sd = 4.46; range = 0–20), respectively. Seventy-nine percent (n = 344) of the sample was tested in English, with the remainder completing the assessment in Spanish. Ninety-seven percent of the sample self-reported their racial status as White and 42% (n = 180) reported their ethnicity as Hispanic, with the majority (n = 171) being of Mexican American origin. Of those participants genotyped (n = 440), 329 (75%) were APOE4 negative and 111 (25%) were APOE4 positive. Demographic characteristics of the sample are presented in Table 2. Estimated mean current arsenic level was 6.33 μg/L (sd = 3.03, range = 2.19–15.26). The 15-year mean arsenic concentrations in Parmer and Cochran County, TX were 3.06 μg/L and 7.39 μg/L, respectively. In both counties, there was less than 2 μg/L variability over any given time period with results remaining very stable over this time period. There were 301 participants with all requisite data for calculation of long-term arsenic exposure at current household; mean long-term exposure was 240.15 μg/L-years (sd = 182.96; range = 2.87–972.83). On average, participants resided in their current residence for 34.12 years (sd = 20.01 years, range = 1–80 years).

Current estimated groundwater arsenic exposure level was significantly associated with poorer scores in language (RBANS Language scores, B(SE) = −0.458 (0.171), p = 0.008), visuospatial skills (CLOX2, B(SE) = −0.118 (0.060), p = 0.048), and executive functioning (CLOX 1 B(SE) = −0.225 (0.080), p = 0.005) (see Table 3). Current arsenic exposure significantly classified cognitive dysfunction (AUC = 0.58, 95% CI = 0.51–0.65, p = 0.03).

Long-term low-level exposure to arsenic was significantly associated with poorer scores in global cognition (MMSE B(SE) = −0.003 (0.001), p = 0.004), visuospatial skills (CLOX 2 B(SE) = −0.001 (0.001), p = 0.038), language (FAS B(SE) = −0.012 (0.004), p = 0.002; RBANS Language B(SE) = −0.005 (0.002), p = 0.017), processing speed (TMTA B(SE) = 0.034 (0.014), p = 0.016), executive functioning (EXIT B(SE) = 0.006 (0.002), p < 0.001), and immediate memory (RBANS Immediate Memory B(SE) = −0.010 (0.003), p = 0.003). Long-term low-level arsenic exposure significantly classified cognitive impairment (AUC = 0 .62, 95% CI = 0.55–0.69, p = 0.001).

With the rapidly growing number of elders world-wide, there is a great need for research examining factors that impact cognition among adults and elders. Prior work has consistently demonstrated the adverse health consequences of high levels of arsenic exposure. As a result, the U.S. acceptable standard of arsenic concentration was reduced in 2001 from 50 μg/L to 10 μg/L. However, as pointed out by the National Research Council, there remains a need for research examining the non-cancer health effects of low-level arsenic exposure [7,8] using longitudinal methodologies. Our current findings suggest that long-term low-level arsenic exposure is detrimental for the cognitive status of adults and elders. Our findings are consistent with prior work linking environmental exposure to arsenic to poorer neuropsychological functioning. Bolla-Wilson and Bleeker [44] evaluated the neurocognitive functioning of a 50-year-old adult following acute exposure to arsenic and documented deficits in learning and memory, which improved over time with no subsequent exposures. Similarly, Wright and colleagues [45] examined the neuropsychological profile of 31 school-aged children residing in Ottawa County, Oklahoma, which contains the Tar Creek Superfund site, and found higher hair arsenic levels to be significantly associated with poorer scores on tests of intelligence and memory. Tsai and colleagues [46] evaluated 49 junior school students and found that higher chronic groundwater arsenic exposure was significantly related to poorer memory and executive functioning (*i.e.*, switching attention). In a sample of 602 school children age 6–8 years, Rosado and colleagues [47] found that higher urinary arsenic levels were significantly associated with poorer visuospatial skills, intelligence, attention and executive functioning. In our study, higher levels of current groundwater arsenic exposure (though still low-level exposure) was related to poorer visuospatial skills, language, and executive functioning. Increased levels of long-term low-level exposure were related to significantly poorer performance in the domains of global cognition, language, executive functioning, as well as memory. The current study is, however, different from all of the previously conducted work in several ways. First, the mean and median values of groundwater exposures in the communities evaluated in this study are below the current U.S. acceptable standard and are therefore reflective of low-level exposure. Second, we examined the impact of exposure on neuropsychological functioning in a community-based sample of rural-dwelling adults and elders rather than school children. Lastly, ours is the first to study the impact of estimated *long-term low-level* arsenic exposure on detailed neuropsychological functioning. Our results are consistent with and extend prior work to adults and elders exposed at lower levels of groundwater arsenic [3].

The consistent finding of arsenic being related to the domains of executive functioning and memory is of critical importance as these are cognitive domains that may change as part of the normal aging process and may even be the earliest manifestations of Alzheimer’s disease [48,49]. This is particularly important for the long-term estimates, which as can be seen from Table 3, are more strongly related with cognitive status. Therefore, it is possible that the impact of low-level arsenic exposure on neuropsychological functioning happens over time, which fits with the developmental course of Alzheimer’s disease. Alzheimer’s disease is a disease of insidious onset with slow progression that begins decades before clinical manifestation. While it has been suggested that research should focus on preventative strategies for Alzheimer’s disease [50], the recent NIH consensus panel suggested that there are no preventative strategies currently available for Alzheimer’s disease, albeit dietary factors were suggested as having promise. The notion of groundwater exposure to arsenic as a risk factor for late-life Alzheimer’s disease offers potential for the first ever population-wide preventative effort aimed at preventing and/or delaying onset of this disease, which could be accomplished by revision of the Safe Drinking Water Act (SDWA).

There are limitations to the current study. One limitation is the cross-sectional nature of the data, which does not allow for inference of causality. Longitudinal assessment of this topic will be conducted through Project FRONTIER as follow-up waves are completed. The key limitation to the current study is the lack of either a biomarker of arsenic exposure (*i.e.*, blood, hair, or nail levels) or direct measurement of the actual water arsenic levels at participant homes. However, the existence of the TWDB data provides an exceptional opportunity to model chronic arsenic exposure and the state can serve as a naturalistic setting for future studies. This is due to the fact that there are several “hot beds” of high arsenic levels across the state, even though the majority of the state is well below the current EPA standard. It is shown that GIS-estimated arsenic concentrations are very close to the measured values, particularly in terms of rank. Future Project FRONTIER studies will examine serum arsenic levels along with current household water levels. We cannot at this point conclude that long-term low-level arsenic consumption through water is causally related to poorer cognition from the current data. However, we can assert that those individuals who have resided for long periods of time in regions that have historically low levels of arsenic in groundwater supplies are at increased risk for cognitive dysfunction. This finding is certainly novel and warrants further investigation.

## 4. Conclusions

Our findings suggest an association between low-level arsenic exposure and neuropsychological functioning, as originally hypothesized. Of particular interest is the association between long-term low-level arsenic exposure and neuropsychological functioning across a broader range of domains than current exposure. Our findings offer the first direct evidence that low level arsenic exposure, extrapolated from current arsenic levels and self report of duration in residence is associated with poorer neuropsychological functioning among community-dwelling adults and elders in the U.S. Further research is needed to investigate this topic and, if cross-validated, this research will provide ample justification for a re-evaluation of current policy related to acceptable groundwater arsenic levels.

## Figures and Tables

**Table 1.** Comparison of GIS-estimated *vs.* measured arsenic concentration in seven wells (μg/L).

Well #	GIS-Estimated	Measured
1	12.9	14.2
2	12.0	14.4
3	11.6	13.3
4	10.5	10.7
5	7.0	5.8
6	6.2	1.6
7	5.8	1.6

**Table 2.** Demographic characteristics.

	Mean (sd)	Range
Age	62.12(12.8)	40–96
Education	10.84 (4.46)	0–20
MMSE	27.54 (2.80)	12–30
Arsenic (μg/L)	6.33 (3.03)	2.19–15.26
Long-Term Arsenic (μg/L-years)	240.15 (182.96)	2.87–972.83
FAS Total	27.93 (12.62)	0–71
Animal Naming	16.35 (5.01)	0–32
TMTA (seconds)	56.73 (34.00)	18–420
TMTB (seconds)	127.32 (77.67)	33–532
RBANS Immediate Memory	41.11 (9.39)	5–61
RBANS Visuospatial	28.80 (6.26)	0–40
RBANS Language	27.36 (5.46)	8–42
RBANS Attention	45.51 (16.72)	9–100
RBANS Delayed Memory	35.76 (9.25)	10–60
EXIT-Total	7.31 (4.77)	0–23

Note: RBANS scores are reflective of raw index scores.

**Table 3.** Arsenic levels impact on neuropsychological functioning.

	**Long-Term Arsenic**		**Current Arsenic**	
	B (SE)	p-value	B (SE)	p-value
MMSE	−0.003 (0.001)	0.004	−0.116 (0.088)	0.191
CLOX 1	−0.001 (0.001)	0.290	−0.225 (0.080)	0.005
CLOX 2	−0.001 (0.001)	0.038	−0.118 (0.060)	0.048
FAS	−0.012 (0.004)	0.002	−0.724 (0.377)	0.056
Animal Naming	−0.002 (0.002)	0.250	−0.138 (0.181)	0.446
TMTA	0.034 (0.014)	0.016	0.986 (1.17)	0.400
TMTB	0.037 (0.029)	0.209	4.23 (2.44)	0.084
RBANS Immediate Memory	−0.010 (0.003)	0.003	0.187 (0.311)	0.547
RBANS Visuospatial	−0.001 (0.002)	0.543	−0.342 (0.211)	0.106
RBANS Language	−0.005 (0.002)	0.017	−0.458 (0.171)	0.008
RBANS Attention	−0.007 (0.005)	0.118	−0.466 (0.423)	0.271
RBANS Delayed Memory	0.001 (0.003)	0.651	0.537 (0.303)	0.077
EXIT Total	0.006 (0.002)	0.000	0.475 (0.139)	0.077

Note: Covariates included age, gender, education, language of administration (English or Spanish), selenium level and APOE4 presence (yes/no); B = unstandardized regression coefficient; SE = standard error.
